# Psychostimulant Use Disorder, an Unmet Therapeutic Goal: Can Modafinil Narrow the Gap?

**DOI:** 10.3389/fnins.2021.656475

**Published:** 2021-05-26

**Authors:** Melinda Hersey, Amanda K. Bacon, Lydia G. Bailey, Mark A. Coggiano, Amy H. Newman, Lorenzo Leggio, Gianluigi Tanda

**Affiliations:** ^1^Medication Development Program, Molecular Targets and Medication Discovery Branch, National Institute on Drug Abuse, Intramural Research Program, National Institutes of Health, Baltimore, MD, United States; ^2^Clinical Psychoneuroendo- crinology and Neuropsychopharmacology Section, Translational Addiction Medicine Branch, National Institute on Drug Abuse Intramural Research Program, National Institutes of Health, Baltimore, MD, United States; ^3^National Institute on Alcohol Abuse and Alcoholism, Division of Intramural Clinical and Biological Research, National Institutes of Health, Bethesda, MD, United States

**Keywords:** cocaine, modafinil, dopamine, dopamine transporter blocker, psychostimulant use disorder, drug abuse and dependence, drug addiction

## Abstract

The number of individuals affected by psychostimulant use disorder (PSUD) has increased rapidly over the last few decades resulting in economic, emotional, and physical burdens on our society. Further compounding this issue is the current lack of clinically approved medications to treat this disorder. The dopamine transporter (DAT) is a common target of psychostimulant actions related to their use and dependence, and the recent availability of atypical DAT inhibitors as a potential therapeutic option has garnered popularity in this research field. Modafinil (MOD), which is approved for clinical use for the treatment of narcolepsy and sleep disorders, blocks DAT just like commonly abused psychostimulants. However, preclinical and clinical studies have shown that it lacks the addictive properties (in both behavioral and neurochemical studies) associated with other abused DAT inhibitors. Clinical availability of MOD has facilitated its off-label use for several psychiatric disorders related to alteration of brain dopamine (DA) systems, including PSUD. In this review, we highlight clinical and preclinical research on MOD and its R-enantiomer, R-MOD, as potential medications for PSUD. Given the complexity of PSUD, we have also reported the effects of MOD on psychostimulant-induced appearance of several symptoms that could intensify the severity of the disease (i.e., sleep disorders and impairment of cognitive functions), besides the potential therapeutic effects of MOD on PSUD.

## Introduction

Psychostimulant use disorder is a complex disease defined by DSM-5 which includes both former (DSM-IV) diagnoses of abuse and dependence on a psychostimulant, such as cocaine or amphetamines. While illicit drugs have long been a societal concern, drug use rates have been growing in recent years. Globally, stimulants such as cocaine and amphetamines are used by approximately 0.35–0.4% and 0.7–0.77% of the population, respectively (Peacock et al., 2018; Farrell et al., 2019). Of these subpopulations, 16% are dependent on cocaine, while 11% are dependent on amphetamines (Farrell et al., 2019). In the United States, it was estimated that about 5.5 million people age 12 and older used cocaine in 2018 (2% of the United States population) (SAMHSA, 2018) and 1.9 million people age 12 and older used METH in 2018 (0.7% of the United States population) (SAMHSA, 2018). A major issue with substance use disorders is the risk of overdose. Recent data show that between 2012 and 2018, drug overdoses involving cocaine more than tripled, and drug overdoses involving abused psychostimulants increased nearly five-fold (Hedegaard et al., 2020).

Classically, the neurobiology underlying PSUD has focused on the neurotransmitter dopamine (DA) for its role in reward processing (Wise and Rompre, 1989; Wise, 2008; Arias-Carrión et al., 2010; Taber et al., 2012). Indeed, commonly abused stimulants exert effects on brain DA levels through their interactions with the neuronal membrane DAT (Das, 1993; Nestler, 2005). Increased DA levels after psychostimulant administration lead to arousal and euphoria, which facilitate the transition from the initial recreational use to continued excessive use, and parallel the potential clinical development of addiction in patients with the most severe form of the disorder (Compton et al., 2018).

The clinical severity of PSUD can be often worsened by medical and mental health comorbidities, e.g., mood and sleep disorders (Mahfoud et al., 2009; Gould, 2010; Torrens and Rossi, 2015). Furthermore, PSUD may be associated with cognitive impairment, which in turn lead to higher treatment dropout rates (Sofuoglu et al., 2013, 2016; Nuijten et al., 2016). These indicate a potential treatment avenue to ameliorate some of the effects of PSUD, which may contribute to increased abstinence rates overall.

Treatment of PSUD relies primarily on behavioral remedies, which may include 12-step facilitation, contingency management, relapse prevention, motivational enhancement therapy, and CBT (for a review, see: Vocci and Montoya, 2009). However, these approaches are time- and resource-intensive and their effect sizes are sub-optimal: integration with effective pharmacotherapies would be likely to improve outcomes and success rates. However, to date there are no approved pharmacologic treatments for PSUD (Phillips et al., 2014). Medications such as antidepressants, DA agonists/partial agonists, mood stabilizers, neuro-protectives, and agonist-like replacement therapy (de Lima et al., 2003; Elkashef et al., 2005; Diana, 2011; Phillips et al., 2014; Jordan et al., 2019) have all been tested with minimal success. The lack of pharmacological treatments for PSUD is a driving force for research toward the development of novel medications.

Among the potential pharmacotherapeutic options for PSUD is MOD, a clinically available medication that inhibits the uptake of DA by blocking DAT (Mignot et al., 1994; Loland et al., 2012). This pharmacological effect is shared with abused psychostimulants but, in spite of that, MOD shows behavioral and neurochemical actions that suggest limited, if any, potential for misuse (Jasinski, 2000; Deroche-Gamonet et al., 2002; Myrick et al., 2004; Food and Drug Administration, 2007; Vosburg et al., 2010; Mereu et al., 2020). Currently, this agent is prescribed for its wake-promoting effects (Czeisler et al., 2005; Kumar, 2008), consistent with its approval for narcolepsy, shift work sleep disorder, and obstructive sleep apnea/hypopnea syndrome. Off-label, MOD has been used for its pro-cognitive effects, especially in patients with cognitive impairment associated with psychiatric disorders (Peñaloza et al., 2013; Turner et al., 2014). During the last two decades, MOD has been tested as a potential medication to treat some of the primary (dependence) and secondary (cognitive and sleep disorders) symptoms of PSUD, representing a potential additional treatment option for selected populations affected by PSUD.

In this review, we will mainly focus on preclinical studies showing how MOD and R-MOD (its R-enantiomer) interact with DAT, the DAergic system, and the reinforcing actions of abused psychostimulants. We will also review clinical studies where MOD efficacy as a potential treatment for PSUD has been evaluated, including for related symptoms such as alteration of sleep and cognitive dysfunction.

## Modafinil Pharmacology Related to PSUD

Modafinil [2-(Diphenylmethyl) sulfinyl acetamide; Alertec, Modavigil, Provigil] and its long-acting, enantiopure form, R-MOD (Nuvigil, Artvigil) (Wong et al., 1999), are clinically available and prescribed as wake-promoting agents for narcolepsy and sleep disorders (Bastoji and Jouvet, 1988; Broughton et al., 1997; US Modafinil in Narcolepsy Multicenter Study Group, 1998, 2000). Early evidence suggested that MOD had a weak, low μM affinity, but relatively good selectivity, for DAT (Mignot et al., 1994), confirmed by more recent studies (Madras et al., 2006; Loland et al., 2012). Thus, the main mechanism of action for MOD appears predominantly driven by actions at neural membrane DATs to stimulate catecholamine neurotransmission (Wisor et al., 2001; Madras et al., 2006). DAT knockout mice were used to confirm the importance of DAT in the mechanism of action of MOD, as studies have found that the pharmacological wake-promoting effects of MOD administration were abolished in those mutant mice (Wisor et al., 2001). Volkow et al. (2009) used PET to show that, after oral administration, MOD (200 to 400 mg) occupies and blocks DAT in the human brain (caudate, NAcc, and putamen). The latter effect was also shown for the enantiomer, R-MOD (Spencer et al., 2010). Further, as a result of the DAT inhibition induced by administration of MOD or R-MOD, increased brain DA levels can be observed in several dopaminergic nerve terminal regions (Ferraro et al., 1996c; Wisor et al., 2001; Volkow et al., 2009; Loland et al., 2012). Further, DAT trafficking could be affected by psychostimulants. Administration of DAT substrates like METH and amphetamine decreases the trafficking of DAT to the cell surface (Saunders et al., 2000; Zahniser and Sorkin, 2009), while DAT inhibitors like cocaine have been shown to increase DAT trafficking to the cell surface (Daws et al., 2002; Little et al., 2002; Zahniser and Sorkin, 2009). Although the effects of MOD administration on DAT trafficking have yet to be fully elucidated, it has been shown that MOD prevents METH-induced decreases in DAT immunoreactivity 6 days after treatment (Raineri et al., 2012).

Beyond DAT, MOD does not show significant affinity for other important pharmacological brain targets. For example, MOD affinity for the NET falls in the 100 μM range (Madras et al., 2006), and it is still unclear if the increases in brain NE levels induced by MOD are the result of its interaction with NET (see for review Mereu et al., 2013). These effects on brain NE levels in PFC and rostro-medial hypothalamus (de Saint Hilaire et al., 2001) could be of interest due to a well-documented role for NE in wakefulness and arousal (reviewed in Mitchell and Weinshenker, 2010). Interestingly, MOD did not show direct activity on trace amine-associated receptor 1 (TAAR1) (Madras et al., 2006), in contrast to amphetamines (Xie and Miller, 2009; Liu et al., 2020). MOD has been shown to have indirect actions on TAAR1 through activation of DAT, which can augment TAAR1 activation (Madras et al., 2006). TAAR1 has been implicated in wakefulness, which represents a predictable effect given the receptor’s ability to modulate the activity of other monoamine systems (Revel et al., 2013; Liu et al., 2020). In a recent report, deletion of TAAR1 receptor in mice did not produce substantial effects on MOD-induced wakefulness as compared to WT mice (Schwartz et al., 2018). In the same report, reductions in MOD-induced gamma-band activity in EEG studies in TAAR1 KO mice were found, and the authors suggest that TAAR1 may regulate neurophysiological factors related cortical and cognitive functions (Schwartz et al., 2018).

Regardless of its affinity for pharmacological targets, MOD has been reported to affect the levels of several neurotransmitters. MOD stimulates brain glutamate levels in the hypothalamus (medial preoptic area and posterior hypothalamus), thalamus (ventromedial and ventrolateral regions), and hippocampus (Ferraro et al., 1997b, 1999), and it has been shown to decrease the levels of GABA in the NAcc, hypothalamus (medial preoptic area and posterior hypothalamus), striatal, and pallidal regions (Ferraro et al., 1996b, 1997a, 1999). MOD induced stimulation in brain serotonin levels in the PFC (Ferraro et al., 2000; de Saint Hilaire et al., 2001), increases in histamine levels and/or activation in the tuberomammillary nucleus and the anterior hypothalamus (Scammell et al., 2000; Ishizuka et al., 2003, 2008), and limited activation of orexin/hypocretin neurons in the perifornical areas and lateral hypothalamus (Chemelli et al., 1999; Scammell et al., 2000; Willie et al., 2005) has also been observed (reviewed in Kumar, 2008; Minzenberg and Carter, 2008; Mereu et al., 2013).

In addition to its effects on neurotransmitter levels, MOD administration affects the induction and inhibition of hepatic cytochrome P450 isoenzymes (Robertson et al., 2000). *In vitro*, MOD competitively inhibits CYP2C19 and suppresses CYP2C9, as well as moderately induces CYP1A2, CYP3A4, and CYP2B6 (Robertson et al., 2000). Pharmacokinetic studies *in vivo* with warfarin and ethinylestradiol, which react with CYP2C9 and CYP3A4 respectively, have not shown the same magnitude of effect as *in vitro* studies (Robertson and Hellriegel, 2003). Through MOD’s induction and inhibition of the P450 isoenzymes, MOD co-administration may decrease or prolong plasma concentrations of other drugs metabolized through these enzymes (Schwartz, 2005). There have been clinical reports of MOD interactions with medications, for example, cyclosporine and clomipramine. Specifically, the immunosuppressive effect of cyclosporine decreased after 200 mg/day MOD, which appeared to be from CYP3A4 induction (for a review, see e.g., Robertson and Hellriegel, 2003). A patient treated with clomipramine was found to lack functional CYP2D6, and the ancillary CYP2C19 pathways inhibited by MOD contributed to increased clomipramine levels in the blood (Robertson and Hellriegel, 2003). MOD also has notable effects as a facilitator of electrotonic coupling in neurons and astroglia through actions at gap junctions (Garcia-Rill et al., 2007; Urbano et al., 2007; Liu et al., 2013; Duchêne et al., 2016; Mereu et al., 2020). In particular, it has been shown that the gap junction inhibitor carbenoxolone blunted the ability of MOD to potentiate self-administration of cocaine in rats (Mereu et al., 2020). These properties are likely important for the agent’s pharmacological actions, as well as interactions with other drugs and biomolecules.

### Modafinil, DAT Inhibition, and Potential Abuse Liability

As a result of inhibition of DAT, it is not surprising that MOD activities could overlap with some of those observed after administration of commonly abused psychostimulants. However, as reported in Table 1, some of its actions seem directed to improve specific symptoms observed in patients with a PSUD diagnosis, i.e., impairments in cognition, sleep, cardiovascular function, and mood disturbances, as well as elevated neuroinflammation. Moreover, MOD fails to display the abuse potential (Jasinski, 2000; Deroche-Gamonet et al., 2002; Myrick et al., 2004; Food and Drug Administration, 2007; Vosburg et al., 2010) or the withdrawal symptoms (Hermant et al., 1991; Myrick et al., 2004) observed with typical psychostimulants. Indeed, to our knowledge, only a very few anecdotical reports of MOD abuse and dependence have been reported in the literature (Kate et al., 2012; Ozturk and Deveci, 2014; Krishnan and Chary, 2015) despite the climbing rates of its non-medical use as a cognitive enhancer in schools and at the workplace (Sharif et al., 2021). Further, important behavioral and neurochemical differences between MOD, or R-MOD, and typical abused psychostimulants have been found in preclinical studies, suggesting they have a unique pharmacological, psychostimulant profile. Taken together, these actions highlight the potential for MOD to reduce the harm associated with the complexity of the symptoms in PSUD. In the next sections, we will briefly highlight some of the pharmacological actions of MOD, i.e., increased wakefulness, improved cognition and cardiovascular function, that could play a potential role in its therapeutic activity against PSUD (summarized in Table 1).

**TABLE 1:** Symptoms related to PSUD and potential therapeutic actions of MOD.

**PSUD symptoms**		**Actions of MOD**	
Recreational use, misuse, and potential for dependence (DEA schedule 1 or 2)	Gawin (1991); Barr et al. (2006)	Low abuse liability (DEA schedule 4)	Jasinski (2000); Deroche-Gamonet et al. (2002); Myrick et al. (2004); Food and Drug Administration (2007); Vosburg et al. (2010)
		Substitute for psychostimulants	Gold and Balster (1996); Reichel and See (2012)
		Decreased cocaine use	Dackis et al. (2005); Hart et al. (2008)
		Decreased nicotine use	Wang et al. (2015)
		Decreased METH use	Shearer et al. (2009); De La Garza et al. (2010)
Cardiovascular issues	Lange and Hillis (2001); Kaye et al. (2007); Duflou (2020)	Regulates heart rate disruptions associated with amphetamines	Makris et al. (2004); De La Garza et al. (2010)
Impaired cognition	Bolla et al. (1999); Nordahl et al. (2003)	Improved attention, concentration, executive function	Pigeau et al. (1995); Minzenberg and Carter (2008); Killgore et al. (2009); Finke et al. (2010); Dean et al. (2011)
Impaired mood	Rounsaville et al. (1991); Salo et al. (2011)	Improved mood	Pigeau et al. (1995)
		Improve depressive illness	Frye et al. (2007)
		Increased/decreased anxiety	Sarnyai et al. (1995); Salo et al. (2011)
Elevated neuroinflammation	Kousik et al. (2012)	Protects against PSUD-related neuroinflammation	Raineri et al. (2012)
Sleep disruptions	Schierenbeck et al. (2008); Hasler et al. (2012)	Wake-promoting agent	Beusterien et al. (1999); Scammell et al. (2000)
		Fatigue reducing	Pigeau et al. (1995)

*METH = methamphetamine; DEA = Drug Enforcement Agency; PSUD = psychostimulant use disorder; MOD = modafinil.*

### Modafinil Interactions With Sleep and Wakefulness Activities

Modafinil was introduced as a wake-promoting agent in the 1990s and approved by the US FDA to treat excessive sleeping (narcolepsy, shift work sleep disorder, obstructive sleep apnea/hypopnea syndrome) (Bastoji and Jouvet, 1988; Broughton et al., 1997; US Modafinil in Narcolepsy Multicenter Study Group, 1998, 2000). In addition to its approved uses, MOD is often used off-label for the treatment of fatigue symptoms in neurological, psychiatric, and excessive fatigue disorders (reviewed in Kumar, 2008). Like amphetamines, the molecular mechanism by which MOD imposes wakefulness has been largely debated. The mechanism is suspected to be linked to the inhibition of monoamine transport by plasma membrane transporters like DAT. In particular, studies have shown that DAT and DA regulation in key areas involved in wakefulness/sleep (i.e., medial preoptic area and posterior hypothalamus) is crucial in the wake-promoting effects of amphetamines and MOD (Jones et al., 1977; Nishino and Mignot, 1997; Nishino et al., 1998; Wisor et al., 2001). Further, researchers have shown that DA activity fluctuates with arousal state (Trulson, 1985). MOD’s effects on other neurotransmitters are also suspected to play a role in the wakefulness-promoting properties of this agent (Boutrel and Koob, 2004). For example, changes in brain NE neurotransmission have been suggested to play a role in wakefulness and arousal (reviewed in Mitchell and Weinshenker, 2010), and MOD stimulation of brain NE levels in PFC and rostro-medial hypothalamus (de Saint Hilaire et al., 2001) could be related to its effect on sleep disturbances. While it is still unclear if NET blockade is the mechanism of action related to MOD stimulation of brain NE levels, some studies have suggested that NET inhibition is less efficacious in the promotion of wakefulness than DAT inhibition (Nishino and Mignot, 1997; Nishino et al., 1998). It is of interest to note that MOD, as highlighted above, interacts with other key neurotransmitters and systems that could play a role in the regulation of sleep, including glutamate, GABA, serotonin, histamine, and orexin/hypocretin (reviewed in Monti, 2013). There is also evidence that MOD increases glutamine synthetase in the rat brain, an enzyme that converts glutamate to glutamine for storage, which may be important for the wakefulness effects of MOD (Touret et al., 1994). The orexin system has a well-established role in sleep-wake regulation (Espana et al., 2001; Sakurai, 2007). MOD administration increases the expression of c-Fos (a marker of neuronal activation) in orexin neurons in the hypothalamus (Chemelli et al., 1999; Scammell et al., 2000). Orexin neuronal projections can activate histamine release in the hypothalamus as well (Huang et al., 2001; Ishizuka et al., 2002). Histamine also has a well-documented role in regulating sleep-wake cycle (Haas et al., 2008). Interestingly, MOD administration produced activation of histaminergic cells (Scammell et al., 2000) but only in the presence of an intact orexin system (Ishizuka et al., 2010). Further, decreases in histamine or loss of histamine neurons blunted MOD-induced increases in locomotion (Ishizuka et al., 2008) as well as the drug’s wake-promoting actions (Yu et al., 2019). On the other hand, orexin-null mice displayed heightened wakefulness following MOD administration compared to wild-type mice (Willie et al., 2005). These findings suggest impaired regulation of the arousal system following removal of orexin, but they also suggest that orexin is not necessarily required for MOD’s wake-promoting actions, or that in those mice possible neuronal adaptations would substitute for removal of orexin.

### Modafinil Interactions With Cognitive Functions

Enhancements in cognitive functions have been reported following MOD administration in rodents and humans (Turner et al., 2003; Minzenberg and Carter, 2008; Cope et al., 2017). MOD produces dose-dependent improvements in working memory (Béracochéa et al., 2001; Ward et al., 2004; Piérard et al., 2006), speed of learning (Béracochéa et al., 2002, 2003; Ward et al., 2004), and sustained attention (Morgan et al., 2007) in animals. In humans, MOD produces similar effects, improving memory and attention (reviewed in Minzenberg and Carter, 2008). Importantly, MOD heightens attention independently of its effects on wakefulness/arousal (Cope et al., 2017). MOD administration elicits changes in activation of brain regions associated with cognition, including the hippocampus (Ferraro et al., 1997b; Shuman et al., 2009; Brandt et al., 2014; Yan et al., 2015) and the PFC (Müller et al., 2004; González et al., 2014, 2018). Thus, improvements in cognitive functions associated with MOD’s actions on the dopaminergic system may underlie those specific changes in DA transmission (reviewed in Minzenberg and Carter, 2008; Mereu et al., 2013), for example in the PFC, that have been recognized for their role in working memory (Sawaguchi and Goldman-Rakic, 1991, 1994). Further, DA receptors can be found on glutamatergic pyramidal cells (Tseng and O’Donnell, 2004) and GABAergic neurons (Tseng and O’Donnell, 2007) in the PFC where they can gate glutamatergic and GABAergic transmission linked to cognition (reviewed in Minzenberg and Carter, 2008). MOD’s effects on brain NE may also affect cognition due to NE’s established role in modulation of cognitive function (reviewed in Chamberlain and Robbins, 2013). Stimulation of NE neurotransmission following MOD administration is also implicated in cognition (Minzenberg and Carter, 2008), while MOD actions on the acetylcholine system have been shown to have effects on learning and memory (reviewed in Mereu et al., 2013). It has also been shown that MOD produced increased motivation, likely by activating D1 receptors (Young and Geyer, 2010). Importantly, MOD is an appealing candidate to target cognitive dysfunction associated with ADHD and psychiatric disorders (Ballon and Feifel, 2006), as well as PSUD. Specifically, treatment with MOD has been shown to improve cognition in PSUD patients (Dean et al., 2011) (see also the “Human studies” sections below).

The pro-cognitive effects of MOD have stimulated a debate about an ethical dilemma and potential concern regarding its rapidly increasing off-label, non-medical use in healthy individuals to improve attention, focus, memory, and cognitive functions (Cakic, 2009; Sahakian and Morein-Zamir, 2011; Peñaloza et al., 2013).

### Modafinil/DAT Inhibition and Inflammation

Additional potential actions of MOD include the ability to act as an anti-inflammatory agent. Specifically, MOD has been shown to reduce neuroinflammation via suppressing inflammatory cytokines (Han et al., 2018), T-cell differentiation (Brandao et al., 2019), monocyte recruitment/activation (Zager et al., 2018), and activation of glial cells (Raineri et al., 2012). This MOD-induced immune activation may be essential for decreasing the neurotoxic and inflammatory consequences of many diseases including PSUD, an exceptionally important effect given that many stimulants are pro-inflammatory in nature. METH administration is marked by increases in TNF-α, IL-1β, and IL-6 expression, as well as elevated microglial activation (Cadet et al., 1994; Lai et al., 2009; Gonçalves et al., 2010). Cocaine has similarly been associated with increases in TNF-α, IL-6, IL-8, activator protein 1 (AP-1), and nuclear factor kappa B (NFκB) (Zhang et al., 1998; Gan et al., 1999; Lee et al., 2001; Dhillon et al., 2008). Nicotine is marked by increases in TNF-α, IL-18, IL-1β, and chemokines, including CCL2, CCL8, and CXC3CL1 (Bradford et al., 2011). Pro-inflammatory agents, such as stimulants, have also been associated with deterioration of the natural obstacle that protects the brain; the blood brain barrier, further magnifying their neurotoxic effects (Czub et al., 2001; Nath et al., 2002). MOD has been shown to counteract the toxic and neuroinflammatory effects of METH in mice (Raineri et al., 2012), but effects against other drugs of abuse have yet to be reported.

Modafinil administration has also been shown to exert effects on histamine, a common marker of inflammation and neurotransmitter involved in sleep/wakefulness (Haas et al., 2008). Using *in vivo* microdialysis, an increased histamine release in the anterior hypothalamus was observed following MOD administration (Ishizuka et al., 2003).

## Preclinical Studies on MOD as a Pharmacotherapeutic Treatment for PSUD

### Neurochemical Studies

In this section, we will review the neurochemistry of MOD as it relates to PSUD. The main pharmacologic activity of MOD is due to its affinity and inhibitory actions at DAT, which result in stimulation of brain extracellular DA levels. DAT and the DA system also play a major role in the abuse liability of psychostimulants. Thus, we will start this section with a brief background about DAT and DA roles in PSUD.

#### DA and DAT, Their Role in Drug Abuse, Dependence, and as Potential Targets for Pharmacotherapy of PSUD

Dopamine’s role in the brain’s reward circuit has been extensively studied (Wise and Rompre, 1989; Di Chiara et al., 1993a, 1998; Wise, 2008; Arias-Carrión et al., 2010; Taber et al., 2012), however its role in drug abuse and dependence is still not fully clarified (Volkow et al., 2011; Wise and Robble, 2020). Following acute administration of drugs of abuse, including central stimulants and depressants, opiates, cannabinoids, and cholinergic agonists, increased levels of extracellular DA have been reported in the brain regions that are the projection fields of dopaminergic neurons, specifically the NAcc and caudate (Di Chiara and Imperato, 1988; Koob, 1992; Pontieri et al., 1995, 1996; Tanda et al., 1997a; Di Chiara et al., 1999). Acute administration of psychostimulants, in particular, has been shown to increase DA levels in a dose dependent manner in the NAcc shell and core, and in the striatum (Di Chiara et al., 1993b; Pontieri et al., 1995; Tanda et al., 1997b). These effects are likely related to the initial positive experience of drug use that could also lead to acquisition of drug-seeking behaviors and to the desire to repeat behaviors that lead to a pleasurable experience (Pettit and Justice, 1989; Woolverton and Johnson, 1992; Koob et al., 1998), but do not account for all neurological aspects of substance use disorder (Salamone et al., 2003; Robinson and Kolb, 2004; Russo et al., 2009; Golden and Russo, 2012). Repeated drug use has been shown to cause synaptic changes, allowing for the development of a different regulation of neurotransmission and other neuronal activities, which is believed to be the driving force behind drug addiction (Thomas et al., 2008; Luscher and Malenka, 2011). Indeed, addictive drugs consistently elicit neurological changes that are indicative of potential targets for better understanding and treating the development of specific patterns of drug use and dependence.

Regulation of expression and trafficking of presynaptic DATs by synaptic DA levels has been proposed as a pharmacological target involved in the development of PSUD (Zahniser and Sorkin, 2004). Indeed, both acute and chronic cocaine exposure increases DAT density in the NAcc and DS (Zahniser and Sorkin, 2004), while other psychostimulants such as amphetamine and METH decrease DAT expression in the same regions (Saunders et al., 2000; Sandoval et al., 2001; Barr et al., 2006; Kahlig et al., 2006). Despite varying levels of transporter presence, a primary result of psychostimulant use is an increase in synaptic DA levels by inhibiting its presynaptic neuronal reuptake or by interacting with the VMAT2, releasing DA into the cytoplasm and then releasing DA into the synapse by reversing its transport direction through DAT (Sulzer et al., 2005; Xie and Miller, 2009; Calipari et al., 2013). The regulation of DAT expression allows the formation of a feedback loop between DAT abundance and psychostimulant presence in the brain (Verma, 2015). The resulting changes in DAT density after drug use perpetuates a need for consistent amounts of the drug to avoid withdrawal and to maintain significant levels of DA and DAT expression.

#### DA and DAT as Potential Pharmacologic Target for the Therapeutic Actions of MOD Against PSUD

The use of MOD as a therapeutic agent for PSUD is largely based on its mechanistic actions that, in part, overlap with those of other abused psychostimulants. For instance, abused psychostimulants increase mesolimbic extracellular DA, often by interacting with DAT (Mortensen and Amara, 2003; Zhu and Reith, 2008), and MOD has been shown to stimulate DA levels in the same dopaminergic areas related to psychostimulant actions (Ferraro et al., 1996c; Zolkowska et al., 2009; Loland et al., 2012; Rowley et al., 2014; Bobak et al., 2016; Mereu et al., 2020). Even though the pharmacological actions of MOD have been mainly explained by its affinity for DAT, its unique psychostimulant profile has been shown to differ from that of typical DAT inhibitors, as shown in behavioral, neurochemical and molecular pharmacology studies (Schmitt and Reith, 2011; Loland et al., 2012; Mereu et al., 2013, 2017, 2020). For example, MOD binding to DAT differs from that of other typical, cocaine-like, DAT blockers (Schmitt and Reith, 2011). In contrast to cocaine, MOD prefers to bind to, or stabilize the DAT protein in a more inward-facing occluded conformation (Schmitt and Reith, 2011; Loland et al., 2012) that still inhibits uptake and results in increases in extracellular DA in the NAcc (Ferraro et al., 1996c; Zolkowska et al., 2009), the NAcc shell (NAS) (Loland et al., 2012; Mereu et al., 2020), and the striatum (Rowley et al., 2014). MOD also increases electrically evoked DA in the DS and VS (Bobak et al., 2016) (summarized in Table 2) like abused psychostimulants (Nisell et al., 1994; Pontieri et al., 1996; Munzar et al., 2004; Kohut et al., 2014). However, while acute administration of MOD (Mereu et al., 2017, 2020) or its enantiomers (Loland et al., 2012; Keighron et al., 2019a, b) increases extracellular NAcc DA levels in rodents, these effects, even at very high doses, elicited a limited stimulation of DA in striatal areas compared to the stimulation elicited by abused psychostimulants (Loland et al., 2012; Mereu et al., 2017, 2020). This limited efficacy of MOD to increase DA levels, as compared to abused psychostimulants, also predicts a limited potential for abuse.

**TABLE 2:** Neurochemical actions of MOD.

**Agent(s)**	**Dose(s), species**	**Effect of MOD**	**References**
MOD	3–300 mg/kg, s.c. RAT	- ↑ Extracellular NAcc DA levels	Ferraro et al. (1996c)
	20–60 mg/kg, i.v.	-↑ Extracellular DA in the NAcc	Zolkowska et al. (2009)
	RAT	- ↓ METH-induced stimulation of NAcc DA levels	
	10–56 mg/kg, i.v. RAT	- ↑ Extracellular NAS DA levels - NSE on cocaine-induced stimulation of NAS DA levels	Mereu et al. (2020)
	10 μg/5 μL, i.c.v. RAT	-↑ Extracellular NAcc DA levels	Murillo-Rodríguez et al. (2007)
	100–600 mg/kg, p.o. RAT	- ↑ Extracellular DA in the striatum and PFC	Rowley et al. (2014)
	30–300 mg/kg, i.p. RAT	- ↑ Electrically evoked DA in the ventral and dorsal striatum	Bobak et al. (2016)
R-MOD	30–100 mg/kg, i.p.	- ↑ Extracellular DA in the NAcc	Wang et al. (2015)
	RAT	- ↓ Nicotine-induced stimulation of NAcc DA levels	
	30–300 mg/kg, i.p. MOUSE	- ↑ Extracellular NAS DA levels	Loland et al. (2012)
	10–32 mg/kg, i.v.	- ↑ Extracellular NAS DA levels	Keighron et al. (2019a)
	MOUSE	- ↑ Electrically evoked NAS DA	
		- ↓ DA clearance rate	
	5–100 mg/kg, i.p.	- ↑ Electrically evoked DA in the NAS	Keighron et al. (2019b)
	MOUSE	- ↓ DA clearance rate	
S-MOD	30–300 mg/kg, i.p. MOUSE	- ↑ Extracellular NAS DA levels	Loland et al. (2012); Mereu et al. (2020)

*NAcc, nucleus accumbens; NAS, nucleus accumbens shell; PFC, prefrontal cortex; METH, methamphetamine; i.p., intraperitoneal; i.v., intravenous; i.c.v, intracerebroventricular injection; s.c., subcutaneous; p.o., oral administration; NSE, not a significant effect; ↑, increase; ↓, decrease.*

Cocaine psychostimulant actions and its abuse liability have been related to its ability to slow DA reuptake by inhibiting DAT and stimulating DA neurotransmission (Wise and Bozarth, 1987; Kuhar et al., 1991). It is interesting to note that administration of MOD (10–32 mg/kg, i.p.) prior to cocaine produced no further increase in extracellular NAS DA levels beyond that produced by cocaine alone (Mereu et al., 2020). This effect varied with the additive effects on DA levels obtained with combinations of cocaine and typical DAT blockers like methylphenidate or WIN 35,428 (Tanda et al., 2009; Mereu et al., 2020), but similar to the effects shown by combinations of cocaine and an atypical DAT blocker like JHW007 (Tanda et al., 2009), suggesting a potential atypical DAT inhibitor effect for MOD in these tests.

Another abused psychostimulant, METH, is transported into DA neurons and its nerve terminals as a DAT substrate, like DA, where it has also been shown to affect the VMAT2 function. As a consequence, decreased vesicular DA concentrations and increased cytoplasmic DA levels result, via reverse transport of DA through DAT (Kahlig and Galli, 2003; Sulzer et al., 2005; Howell and Kimmel, 2008), resulting in dramatic increases in extracellular DA levels and robust stimulation of behavioral activities (Munzar et al., 2004). When administered prior to METH, MOD significantly attenuated the stimulatory effects of METH on extracellular NAcc DA levels (see Table 2) (Zolkowska et al., 2009). This effect suggests the possibility that blockade of DAT by MOD pretreatment could affect the ability of METH to be transported by DAT as its substrate into the DA nerve terminal, thus reducing its ability to enhance extracellular DA levels. Reducing the dopaminergic effects of METH could play a role in the therapeutic effects shown by MOD in some preclinical behavioral reports and in clinical studies on METH dependent subjects.

Nicotine, the key addictive component in tobacco, exerts indirect actions on DAT. Voltammetry studies revealed that nicotine slows DA clearance (Hart and Ksir, 1996), in addition to nicotine’s actions in modulating dopaminergic transmission via activation of nicotinic acetylcholine receptors on DA neurons (Clarke and Pert, 1985; Picciotto et al., 1998; Laviolette and Van Der Kooy, 2004). When administered prior to nicotine, MOD produced a reduction in nicotine-induced stimulation of extracellular NAcc DA levels (see Table 2) (Wang et al., 2015).

These preclinical actions of MOD as an atypical DAT inhibitor suggest a strong potential for its therapeutic use in PSUDs (see Table 2).

#### Modulation of Brain Glutamate Levels by MOD Plays a Role in Its Therapeutic Actions on PSUD

The excitatory neurotransmitter, glutamate, has long been associated with many brain physiological functions and brain diseases including addiction (Meldrum, 2000; Kalivas, 2009). Interestingly, the effects of MOD administration on glutamate levels varies by brain region (reviewed in Gerrard and Malcolm, 2007; Mereu et al., 2013). It is predicted that this could be due, in part, to corresponding activation/inactivation of the inhibitory neurotransmitter, GABA. MOD produced increases in glutamate in the medial preoptic areas (Ferraro et al., 1996b), posterior hypothalamus (Ferraro et al., 1996b), thalamus (Ferraro et al., 1997a), hippocampus (Ferraro et al., 1997a), and striatum (Ferraro et al., 1996a, 1998). It was only at high does (300 mg/kg MOD) that increases in glutamate were observed in the substantia nigra or the pallidum (Ferraro et al., 1998). MOD also shows agonist activity at some glutamate receptors (group II metabotropic; mGlu2/3) (Tahsili-Fahadan et al., 2010), although this is likely not due to direct receptor activation. Behaviorally, the impaired reinstatement of extinguished CPP for opiates following MOD administration was blunted with an mGlu2/3 antagonist pretreatment (Tahsili-Fahadan et al., 2010). Neurochemically, cystine-glutamate exchange or voltage dependent calcium channel antagonist administration blocked increases in glutamate in the NAcc following MOD, in rats chronically trained to self-administer cocaine (Mahler et al., 2014).

The effects of MOD on glutamate can be directly linked to many of the agent’s biological effects. For example, MOD-produced increases in synaptic plasticity and long-term potentiation of glutamatergic connections to orexin neurons in the lateral hypothalamus is linked to improved wakefulness and cognition (Rao et al., 2007), but it is also linked to drug reinforced behaviors (Boutrel et al., 2013).

### Effects of MOD on Behavioral Models of PSUD

Herein, we will review animal preclinical data on behavioral tests, mainly in rodents, used to model specific aspects of human substance use disorders, especially PSUD. Importantly, we will compare results from reports analyzing the effects of psychostimulants alone, MOD alone, and MOD in combination with psychostimulants, as summarized in Table 3.

**TABLE 3:** MOD effects on preclinical behavioral animal models related to PSUD.

**Behavioral test**	**Agent(s), dose(s), species**	**Behavioral effect of MOD**	**References**
Locomotion	MOD, 20–256 mg/kg, i.v. RAT	- ↑ Locomotion and stereotyped movements	Zolkowska et al. (2009)
	MOD, 32–256 mg/kg i.p. RAT	- ↑ Locomotion and stereotyped movements	Chang et al. (2010)
	MOD, 50–75 mg/kg/day p.o. for 30 days RAT	- ↑ Locomotion	Alam and Choudhary (2018)
	MOD, 50–75 mg/kg/day p.o. for 30 days + haloperidol 1 mg/kg i.p. RAT	- Haloperidol ↓ MOD induced locomotion	Alam and Choudhary (2018)
	MOD, 75–150 mg/kg p.o. MOUSE	- ↑ Locomotion and stereotyped movements	Paterson et al. (2010)
	MOD 16–80 mg/kg i.p. MOUSE	- ↑ Locomotion and stereotyped movements	Wuo-Silva et al. (2016, 2019)
	MOD 32–128 mg/k i.p. MOUSE	- ↑ Exploration	Young et al. (2011)
	MOD 64–300 mg/kg i.p. MOUSE	- ↑ Locomotion	Wuo-Silva et al. (2011)
	MOD 64 mg/kg i.p. + sulpiride 25–100 mg/kg i.p. MOUSE	- Low dose sulpiride ↑ and high dose sulpiride ↓MOD induced locomotion	Wuo-Silva et al. (2019)
	MOD 0.75 and 75 mg/kg i.p. + cocaine 15 mg/kg i.p. MOUSE	- NSE effect on locomotion - NSE on cocaine-induced locomotion	Shuman et al. (2012)
	R-MOD 10–30 mg/kg i.p. RAT	- ↑ Locomotion	Zhang et al. (2017)
	R-MOD 30–300 mg/kg i.p. RAT	- ↑ Time awake - Low dose ↑ locomotion	Wisor et al. (2006)
	MOD 20 mg/kg i.v. + METH 0.3 mg/kg i.v. RAT	- ↓ METH-induced locomotion	Zolkowska et al. (2009)
	MOD 3–10 mg/kg i.v. RHESUS MONKEY	- ↑ Nighttime locomotion	Andersen et al. (2010)
Behavioral sensitization	MOD 32–256 mg/kg i.p. RAT	- Induced behavioral sensitization	Chang et al. (2010)
	MOD 16–64 mg/kg i.p. MOUSE	- Induced behavioral sensitization	Wuo-Silva et al. (2016, 2019)
	MOD 64–300 mg/kg i.p. MOUSE	- Induced behavioral sensitization	Wuo-Silva et al. (2011)
	MOD 64–300 mg/kg i.p. + cocaine 15 mg/kg i.p. MOUSE	- Induced bidirectional behavioral sensitization	Wuo-Silva et al. (2011)
	MOD 50 mg/kg i.p. MOUSE	- Induced behavioral sensitization	da Costa Soeiro et al. (2012)
	MOD 75 mg/kg/day p.o. RAT	- Restraint ↑ MOD-induced locomotor sensitization	
	MOD 64 mg/kg i.p. + SCH23390 0.003 mg/kg i.p. or sulpiride 50 mg/kg i.p. MOUSE	- SCH23390 and sulpiride ↓ MOD-induced rapid-onset sensitization	Wuo-Silva et al. (2019)
	MOD 75 mg/kg p.o. MOUSE	- Did not induce locomotor sensitization	Paterson et al. (2010)
	MOD 75 mg/kg i.p. MOUSE	- Did not induce locomotor sensitization	Shuman et al. (2012)
	MOD 64 mg/kg i.p. + cocaine 20 mg/kg i.p. MOUSE	- Induced bidirectional behavioral sensitization	Wuo-Silva et al. (2016)
	MOD 50 mg/kg i.p. + METH 1 mg/kg i.p. MOUSE	- Induced cross-sensitization	da Costa Soeiro et al. (2012)
	MOD 75 mg/kg i.p. + cocaine 15 mg/kg i.p. MOUSE	- Induced cross-sensitization	Shuman et al. (2012)
Conditioned place preference	MOD 32–300 mg/kg i.p. RAT	- Did not induce CPP	Deroche-Gamonet et al. (2002); Tahsili-Fahadan et al. (2010)
	MOD 64 mg/kg p.o. RAT	- Did not induce CPP	Quisenberry et al. (2013)
	MOD 125 mg/kg i.p. MOUSE	- Induced CPP	Nguyen et al. (2011)
	MOD 64–300 mg/kg i.p. MOUSE	- Induced CPP	Wuo-Silva et al. (2011)
	MOD 0.75 and 75 mg/kg i.p. + cocaine 15 mg/kg i.p. MOUSE	- Induced CPP - NSE on cocaine induced CPP	Shuman et al. (2012)
	MOD 128 mg/kg i.p. + cocaine 20 mg/kg i.p. RAT	- Reinstated cocaine CPP	Bernardi et al. (2009)
	MOD 300 mg/kg i.p. + morphine 8–16 mg/kg i.p. RAT	- Did not reinstate morphine CPP	Tahsili-Fahadan et al. (2010)
Self-administration	MOD 0.28–1.7 mg/kg/inj i.v. RAT	- Did not induce self-administration	Deroche-Gamonet et al. (2002)
	MOD 0.1–10 mg/kg/inj i.v. RAT	- Did not maintain self-administration in cocaine-trained rats	Mereu et al. (2020).
	MOD 10–32 mg/kg i.p. + cocaine 0.03–1 mg/kg/inj RAT	- ↑ Cocaine self-administration for lowest dose of cocaine	Mereu et al. (2020).
	MOD 32–128 mg/kg i.p. + cocaine 0.25–1 mg/kg/inj RAT	- NSE on cocaine self-administration	Deroche-Gamonet et al. (2002)
	R-MOD 10–100 mg/kg	- NSE on cocaine self-administration	Zhang et al. (2017)
	i.p. + cocaine 0.5–1 mg/kg/inj i.v. RAT	- ↓ Reinstatement of cocaine seeking at high doses	
	R-MOD/S-MOD 30–100 mg/kg i.p. + nicotine 7.5–60 μg/kg/inj i.v. RAT	- R-MOD ↓ nicotine self-administration - S-MOD ↓ nicotine self-administration at high doses	Wang et al. (2015)
	MOD 30–300 mg/kg i.p. + METH 20 μg/50 μL bolus i.v. inj RAT	- ↓ METH self-administration	Reichel and See (2012)
	R-MOD 100 mg/kg i.p. + METH 0.05 mg/kg/inj i.v.	- ↓ METH self-administration when given 1-h access to METH	Tunstall et al. (2018)
	RAT	- NSE on self-administration when given 6-h access to METH	
	MOD 30–300 mg/kg i.p. + METH	- Did not reinstate METH self-administration	Reichel and See (2010, 2012); Holtz et al. (2012)
	20 μg/50 μL bolus inj or 0.05 mg/kg/inj i.v. RAT	- ↓ Cue, drug, and context induced reinstatement	
	MOD 32 mg/kg/day i.v. for 7–10 days + cocaine 0.001–0.1 mg/kg/inj i.v. RHESUS MONKEY	- MOD ↓ cocaine self-administration	Newman et al. (2010)
	MOD 32–56 mg/kg/day i.v. after extinction of cocaine-maintained self-administration RHESUS MONKEY	- MOD reinstated cocaine self-administration behavior	Newman et al. (2010)
	MOD 0.03–0.3 mg/kg i.v., cocaine 0.02–0.05 mg/kg/inj i.v. RHESUS MONKEY	- ↑ Responding in cocaine-trained subjects switched to MOD	Gold and Balster (1996)
	MOD 3–10 mg/kg i.v. + cocaine 0.1 mg/kg/inj i.v. RHESUS MONKEY	- High dose induced reinstatement	Andersen et al. (2010)
Intracranial self-stimulation	MOD 20–600 mg/kg p.o. RAT	- Facilitate ICSS	Lazenka and Negus (2017)
	MOD 50–150 mg/kg i.p. RAT	- Facilitate ICSS	Burrows et al. (2015)
	R-MOD 50–150 mg/kg i.p. RAT	- Trend toward facilitating ICSS	Burrows et al. (2015)
Drug discrimination	MOD 3–300 mg/kg i.p., cocaine-trained 10 mg/kg i.p. RATS	- 67% cocaine substitution at doses above 100 mg/kg	Gold and Balster (1996); Paterson et al. (2010)
	MOD 30–300 mg/kg i.p., cocaine-trained 10 mg/kg i.p. RAT	- Full substitution at 300 mg/kg	Paterson et al. (2010)
	MOD 10–100 mg/kg i.p., cocaine-trained 10 mg/kg i.p. MICE	- Substitution approached 80% at MOD 56–100 mg/kg administered 5 min before testing, and 100% when administered 60 min before testing	Mereu et al. (2017)
	R-MOD/S-MOD 10–100 mg/kg i.p., cocaine-trained 10 mg/kg i.p. MICE	- Full substitution	Loland et al. (2012)
	MOD 3.2–32 mg/kg i.m., cocaine-trained 0.18–0.4 mg/kg i.m. RHESUS MONKEYS	- 75% substitution	Newman et al. (2010)
Cognitive function	MOD 64 mg/kg p.o. + phencyclidine 5 mg/kg i.p. twice daily for 7 days RAT	- Improved phencyclidine-induced object recognition memory deficits	Redrobe et al. (2010)
	MOD 30–90 mg/kg i.p. + METH 1 mg/kg s.c. for 7 days MOUSE	- Improved METH induced object recognition memory deficits	González et al. (2014)
	MOD 100 mg/kg i.p. + METH 4 mg/kg i.p. 2 h apart, 4 times/day MOUSE	- Improved METH induced object recognition memory deficits	Reichel et al. (2014)
	MOD 50–75 mg/kg/day p.o. for 30 days RAT	- ↑ Memory on Morris Water Maze and passive avoidance task	Alam and Choudhary (2018)
	MOD 50–75 mg/kg/day p.o. for 30 days + haloperidol 1 mg/kg i.p. RAT	- Haloperidol ↓ MOD effects on Morris Water Maze and on passive avoidance task	Alam and Choudhary (2018)
	MOD 0.75–75 mg/kg i.p. MOUSE	- ↑ Learning rate and memory on Morris Water Maze - Low dose ↑ memory of contextual fear conditioning - High dose ↓ memory of contextual fear conditioning - NSE on cued conditioning	Shuman et al. (2009)
	MOD 0.75–75 mg/kg i.p. RAT	- NSE on memory of naïve subjects	Garcia et al. (2013)
	MOD 64 mg/kg p.o. + 20% alcohol (4–6 g/kg/day prenatally, 21 days)	- ↓ Impulsive responses in healthy and prenatal alcohol-treated rats	Heyer-Osorno and Juárez (2020)
	RAT	- ↓ Response latency in prenatal alcohol-treated rats only.	
	MOD 32–64 mg/kg i.p. for 5 days MOUSE	- ↑ Speed of learning - NSE on spatial working memory	Béracochéa et al. (2002)
	MOD 64 mg/kg i.p. for 15 days	- NSE on working memory errors	Burgos et al. (2010)
	RAT	- ↓ Long-term memory errors	
		- ↓ Successes in learning task	
	R-MOD 1–10 mg/kg i.p. + DMSO 1 mL/kg i.p. RAT	- Rescued DMSO-induced deficits in hole-board task	Shanmugasundaram et al. (2017)
		- NSE on normal functioning subjects	
	MOD 32–64 mg/kg i.p. MOUSE	- ↑ Speed of learning	Béracochéa et al. (2003)
	MOD 30–100 mg/kg i.p. RAT	- ↑ Speed of learning at high doses	Ward et al. (2004)
	MOD 8–64 mg/kg p.o.	- ↑ Visual attention	Morgan et al. (2007)
	RAT	- ↑ Impulse control	
		- ↓ Premature responses	
		- NSE on visual discrimination performance	
	MOD 32–128 mg/kg p.o.	- NSE on attentional performance	Waters et al. (2005)
	RAT	- NSE on deficits induced by parametric or pharmacological manipulations	
		- ↑ Premature responding at high doses	
	MOD 3–100 mg/kg i.p. RAT	- ↓ Reaction time in subjects with slow baseline reaction times	Eagle et al. (2007)
		- NSE on subjects with fast baseline reaction time	

*MOD, modafinil; METH, methamphetamine; CPP, conditioned place preference; ICSS, intracranial self-stimulation; i.p., intraperitoneal; i.v., intravenous; s.c., subcutaneous; p.o., oral administration; i.m., intramuscular; NSE, not a significant effect; ↑, increase; ↓, decrease.*

#### Locomotion, Stereotypy, and Behavioral Sensitization

Acute administration of psychostimulant drugs of abuse generally produces a dose-dependent stimulation of exploratory behaviors, including locomotion and stereotyped movements in rodents (Sahakian et al., 1975). Repeated administration of psychostimulants might result in behavioral sensitization (Kalivas and Duffy, 1993; Mereu et al., 2015), a phenomena related to neurobiological adaptations (Ghasemzadeh et al., 2009; Bowers et al., 2010), which lead to a heightened behavioral response to a psychostimulant. The potential of novel drugs to cause sensitization can be indicative of their potential neurological long-term effects that could be related to the development of drug dependence (Kauer and Malenka, 2007).

Modafinil administered alone induced dose-dependent changes in locomotion and stereotyped movements in rats (Zolkowska et al., 2009; Chang et al., 2010; Alam and Choudhary, 2018) and mice (Paterson et al., 2010; Wuo-Silva et al., 2011, 2016; Young et al., 2011), with similar results found in response to R-MOD (Zhang et al., 2017). However, a report by Shuman et al. (2012) found no significant change in locomotion in mice treated with both low and high doses of MOD (Shuman et al., 2012). In rhesus monkeys, nighttime locomotion increased, but daytime locomotion had no significant effect (Andersen et al., 2010), calling into question whether the behaviors measured in these assays are due to the same mechanisms as psychostimulant drugs, or if it is a by-product of the primary wake inducing effects of MOD (Chang et al., 2010). In another report, when locomotion was tested relative to time spent awake in rats, the time awake increased, but locomotor activity only increased for the lowest dose administered (30 mg/kg) (Wisor et al., 2006).

The locomotor activating effects of MOD have also been tested in combination with several psychiatric medications and abused psychostimulants that affect brain neurotransmission at different levels. Haloperidol, a DA D2 receptor antagonist and a commonly prescribed antipsychotic medication, decreased MOD induced locomotion in rats (Alam and Choudhary, 2018), indicating a potential interaction between MOD-induced stimulation of DA levels by blockade of DAT, and inhibition of DA transmission due to blockade of DA D2 receptors by haloperidol. Further, these effects suggest the potential interactions of medications for mental disorders and addiction, which are often found comorbidly. A pretreatment with MOD did not produce significant alteration in cocaine-induced locomotion in mice (Shuman et al., 2012), but MOD significantly decreased METH induced locomotion in rats (Zolkowska et al., 2009), indicating a lack of compounding effects on locomotor activities of MOD in the latter report, which could be dependent on differences in the specific mechanisms of action between different stimulants: cocaine is a DAT blocker, while METH is a DAT substrate and a blocker of the vesicular VMAT2 transporter.

It has been reported that repeated MOD exposure in rats (Chang et al., 2010) and mice (Paterson et al., 2010; Wuo-Silva et al., 2011) would induce behavioral sensitization of locomotion and stereotyped movements, which is further enhanced by exposure to stress (Alam and Chaudhary, 2020). Also, clear individual differences in responses of mice to MOD-induced sensitization have been found (da Costa Soeiro et al., 2012), indicating the importance of better understanding how these differences may lead to individualized treatment. Rapid-onset sensitization was decreased by DA antagonists SCH23390 and sulpiride (Wuo-Silva et al., 2019), and behavioral cross-sensitization was induced between MOD and apomorphine, a direct DA agonist (Chang et al., 2010). MOD administered with cocaine (Wuo-Silva et al., 2011, 2016; Shuman et al., 2012) or METH (da Costa Soeiro et al., 2012) also caused bidirectional sensitization in mice, indicating similar neurological effects of these drugs. While these results require further validation, they may indicate possible neuronal plasticity, which for some drugs has been suggested to have a role in their dependence producing actions (Kauer and Malenka, 2007).

#### Conditioned Place Preference

Drug CPP paradigms consist of classically conditioning an animal to associate a contextually unique location (chamber) with administration of a drug reinforcer, while a different chamber is associated with administration of the reinforcer’s vehicle. After training, animals are given the opportunity to freely explore the distinct locations previously associated with administration of the reinforcer or its vehicle. Assessing the difference in time spent by animals in the two chambers would provide an index of their preference (potentially drug-seeking behavior), indifference, or even aversion toward the chamber associated with the reinforcer (Tzschentke, 2007). Induction of CPP can be obtained by administration of specific doses of drugs of abuse, for example psychostimulants, such as cocaine (Mueller and Stewart, 2000; Itzhak and Martin, 2002) and METH (Itzhak and Martin, 2002), but can also be obtained through illicit drugs (Liu et al., 2008) and other natural reinforcers such as palatable foods (Velázquez-Sánchez et al., 2015). Therefore, CPP is a common preclinical assay that could be used to assess the potential pleasurable effects of a novel medication and to evaluate its potential for abuse.

Modafinil administered alone was unable to induce CPP in rats when administered orally (Deroche-Gamonet et al., 2002), or by intraperitoneal injection (Tahsili-Fahadan et al., 2010; Quisenberry et al., 2013), in contrast with results found in mice (Nguyen et al., 2011; Wuo-Silva et al., 2011; Shuman et al., 2012). These results indicate a minimal, if any, pleasurable effect of MOD, resulting in a low abuse liability for naïve subjects. However, the results in mice indicate a potential species difference, thus further investigation into various model species is required to thoroughly understand the effects of MOD.

The CPP assay can also be applied to understand whether MOD can reinstate seeking of the pleasurable effects of an extinguished behavioral response to a drug of abuse (Napier et al., 2013). Practically, the chamber previously associated with administration of the reinforcer is no longer associated to it, leading the subjects to forget the learned association and return to spending equal amounts of time in both chambers. Reinstatement of CPP occurs quickly following a single administration of the reinforcer. Administration of MOD alone has been shown to reinstate cocaine induced CPP in rats (Bernardi et al., 2009). In contrast to psychostimulants, opioid CPP is not reinstated by MOD treatment (Tahsili-Fahadan et al., 2010). These studies indicate a potential relapse inducing effect of MOD, which may be detrimental to PSUD target subjects. Further investigation is required to determine the varying effects of MOD on reinstatement of drug seeking for different drugs of abuse.

#### Self-Administration

The abuse potential of a substance can be assessed by using animal models of self-administration behavior, which could also model the transition from sampling or recreational use of a substance to its compulsive intake (Ator and Griffiths, 2003; Edwards and Koob, 2013). Compulsive self-administration behavior in animals has been observed under specific experimental operational conditions when selected doses of psychostimulant drugs of abuse such as cocaine or amphetamines were made available (Deneau et al., 1969). In these models, the rate at which subjects would self-administer a substance could indicate the potential for abuse of a novel medication.

Modafinil (0.28–1.7 mg/kg/inj) (Deroche-Gamonet et al., 2002), alone did not promote intravenous self-administration behavior in naïve rats, indicating a lack of MOD reinforcing effects at the doses tested. Further, MOD alone (0.1–10 mg/kg/inj) did not maintain self-administration behavior in rats previously trained with intravenous doses of cocaine (Mereu et al., 2020), and similarly R-MOD was not self-administered in rats trained with nicotine (Wang et al., 2015). However, administration of MOD increased behavioral response rates for at least one dose for each subject in Rhesus monkeys previously trained to self-administer cocaine (Gold and Balster, 1996). This contradiction may be due to species differences or other procedural variables and requires further investigation.

Acute MOD treatment prior to psychostimulant self-administration sessions might indicate whether MOD would affect the reinforcing effects of those drugs. MOD or R-MOD pretreatment did not affect intravenous cocaine self-administration in rats (Deroche-Gamonet et al., 2002; Zhang et al., 2017). In contrast, a more recent study showed increased cocaine self-administration behavior at low cocaine doses (Mereu et al., 2020). Such effect was surprisingly not accompanied by enhancement of cocaine-induced stimulation of NAS DA levels, but it was reversed by pretreatments with carbenoxolone, an inhibitor of electrotonic coupling (Mereu et al., 2020). In rats, R-MOD has been shown to decrease METH (Tunstall et al., 2018) and nicotine self-administration behavior (Wang et al., 2015). Moreover, when MOD was administered chronically, it decreased cocaine self-administration responding in Rhesus monkeys (Newman et al., 2010).

After animals acquire and maintain self-administration behavior induced by abused psychostimulants, these behaviors can be extinguished by stopping drug-injections or eliminating conditioned stimuli associated with the availability or the injection of the drug. After extinction, it has been shown that non-contingent injections of the training drug or reintroduction of its associated cues can reinstate the operant behavior required to deliver the drug, suggesting a potential for relapse. These procedures could also assess the potential effects of test compounds, administered alone or as a pre-treatment, on the likelihood of relapse. Using these procedures, MOD administered alone, either acutely (Reichel and See, 2010; Holtz et al., 2012) or chronically (Reichel and See, 2012), did not reinstate behavior in rats initially trained to self-administer METH. Similar results were found with administration of R-MOD (Wang et al., 2015). However, in rhesus monkeys previously trained to self-administer cocaine, a high dose (10 mg/kg) induced reinstatement of cocaine responding (Andersen et al., 2010), which may indicate a species difference could be a factor in the obtained results. In METH-primed reinstatement tests, both acute (Reichel and See, 2010) and chronic (Reichel and See, 2012) MOD pretreatments attenuated reinstatement of drug-seeking behavior in both male and female rats (Holtz et al., 2012). MOD pretreatments did not significantly modify likelihood for reinstatement of cocaine self-administration behavior in rats (Deroche-Gamonet et al., 2002), but R-MOD reduced cocaine seeking at high doses (Zhang et al., 2017). Additionally, in rats, R-MOD pretreatment reduced nicotine-induced reinstatement of self-administration behavior (Wang et al., 2015). These results indicate that, in contrast to abused psychostimulants, MOD and R-MOD do not induce self-administration behavior, suggesting limited, if any, abuse liability. Also, they may diminish the potential for abuse of psychostimulants or reduce the drive to obtain them, and, finally, attenuate drug-induced reinstatement of drug seeking behaviors, suggesting a potential therapeutic effect in the prevention of relapse to drug use.

#### Intracranial Self-Stimulation

Intracranial self-stimulation is another indicator of the potential abuse liability of a substance. In this procedure, electrodes are placed in the medial forebrain bundle, and electrical stimulation is given when the subject performs the required operant task, for example nose-poking or pressing a lever. In comparison to self-administration studies, where the drug itself acts as the reinforcer, the electrical stimulation is the reinforcer in ICSS studies, allowing the assessment of whether the drug causes increased sensitivity to rewarding stimuli by altering the self-stimulation rates (Negus and Miller, 2014). Cocaine, METH, and other monoamine releasers have been found to facilitate ICSS (Bauer et al., 2013; Negus and Miller, 2014) with a correlation between facilitation rates and DA selectivity (Bauer et al., 2013; Negus and Miller, 2014), further implicating DA and DAT in the rewarding effects of these drugs.

Modafinil has been shown to facilitate ICSS responses in rats when administered orally (Lazenka and Negus, 2017) and intraperitoneally (Burrows et al., 2015). R-MOD shows a trend toward ICSS facilitation at high doses (150 mg/kg) in rats, without reaching significance (Burrows et al., 2015). However, when compared with commonly abused psychostimulants, such as methylphenidate or cocaine, MOD shows significant changes in ICSS rates only when administered at very high doses, while abused drugs show effects at significantly lower doses (Burrows et al., 2015; Lazenka and Negus, 2017). These dose differences may indicate that MOD abuse liability, if any, might require specific conditions, including very high doses, as compared to commonly abused psychostimulants. Indeed, MOD shows very low, if any, abuse liability in humans, and the benefits offered by MOD treatment against PSUD seem to outweigh the possibility of dependence.

#### Drug Discrimination

Administration of drugs, especially those abused by humans, would induce specific interoceptive stimuli that could be perceived and recognized by human subjects as well as animals (Kamien et al., 1993). The ability of subjects to discriminate between interoceptive stimuli elicited by a specific drug and those elicited by the drug’s vehicle could be assessed in drug discrimination procedures (Porter et al., 2018). Indeed, the presence or absence of the drug stimulus could result in different operant responses, for example pressing a lever associated to the drug stimulus or that associated to the drug vehicle. Correct responses are usually rewarded with delivery of food pellets. After training with a specific drug, tests can be performed with administration of, for example, novel compounds. It is important to note that drugs belonging to the same pharmacological class (i.e., opioids, cannabinoids, psychostimulants) usually share a common discriminative stimulus specific for their drug class. Thus, while the drug-discrimination procedure does not measure the reinforcing/rewarding effects of drugs of abuse, similarities between subjective effects of a known abused psychostimulant and novel compounds might suggest their potential for abuse (Katz and Goldberg, 1988; Berquist and Fantegrossi, 2018). Thus, several drug-discrimination studies have tested the possibility that administration of MOD produced subjective effects similar to the discriminative stimulus effects of cocaine.

Modafinil doses below 100 mg/kg produced saline only responses when administered 30 min prior to testing, and higher doses partially substituted for cocaine in rats (Gold and Balster, 1996), but later studies found full cocaine substitution (Paterson et al., 2010). In Rhesus monkeys, MOD dose dependently substituted for cocaine in three of four animals at the highest doses when administered immediately prior to testing (Newman et al., 2010) and in mice, MOD fully substituted for cocaine (Loland et al., 2012; Mereu et al., 2017) when administered 5 or 60 min prior to testing. These results indicate that the subjective effects of MOD are similar to those of cocaine. However, there was a significant difference in potency for those effects, and MOD was found about 10 (Loland et al., 2012; Mereu et al., 2017) to 25 times less potent than cocaine (Gold and Balster, 1996). Further, MOD discrimination responses in rats were lower than that of ephedrine, a common over-the-counter decongestant and bronchodilator (Gold and Balster, 1996). These findings might indicate that high doses of MOD and R-MOD could have abuse potential, but the lower doses which would aid in reducing the likelihood of relapse have little abuse potential, as shown by lack of consistent reinforcing effects in the self-administration studies above.

#### Behavioral Tests Related to Cognitive Functions

Cognitive impairments, such as memory deficits, decision making abilities, and learning rates are a potential concern as a consequence of persistent psychostimulant use (Block et al., 2002). While acute administration of psychostimulants has been found to positively affect cognitive functioning when given immediately prior to testing (Grilly, 2000; Del Olmo et al., 2007), long-term, repeated exposure to these drugs may produce detrimental cognitive effects. For example, in animal models, impairment of cognitive function has been reported in response to chronic administration of METH (Rogers et al., 2008) and cocaine (García-Pardo et al., 2017), among others (Marston et al., 1999; Dalley et al., 2005). MOD has been found to reverse some of the impairments induced by phencyclidine in rats (Redrobe et al., 2010), and by METH in rats and mice (González et al., 2014; Reichel et al., 2014). However, it has been reported that MOD administration had no effect on the object recognition of animals not pretreated with psychostimulants (Reichel et al., 2014), or spatial memory acquisition in rats not treated with DMSO (Shanmugasundaram et al., 2017), indicating a potential restoration of the cognitive impairments induced by drugs of abuse.

Modafinil has also been shown to improve decision making skills by decreasing impulsive responses in rats (Heyer-Osorno and Juárez, 2020), and both acute and chronic administration increases learning and memory abilities in mice (Béracochéa et al., 2002, 2003; Shuman et al., 2009) and rats (Ward et al., 2004; Morgan et al., 2007; Shuman et al., 2009). However, chronic MOD decreased long-term visuo-spatial memory errors, but increased operant conditioning learning errors, indicating an overall benefit for hippocampus dependent tasks in rats (Burgos et al., 2010). in a different study, an enhanced hippocampus dependent memory performance was reported after low doses of MOD (0.75 mg/kg), but not high doses (75 mg/kg), indicating a bell-shaped response curve (Shuman et al., 2009). Further, no effects on impulsive response rates were reported in healthy rats (Waters et al., 2005), however, these findings were explained later when improvements on a response rate task were only present in subjects previously showing slow or impaired response rates (Eagle et al., 2007). In general, these findings indicate that MOD has the potential to enhance cognitive abilities, especially when treating drug of abuse induced impairments, which may influence treatment engagement and likelihood of relapse in PSUD patients (Sofuoglu et al., 2013; Nuijten et al., 2016).

## Human Studies on MOD as a Potential Pharmacotherapy for PSUD

Modafinil has shown therapeutic efficacy for treatment of individuals affected by narcolepsy and sleep disorders (Czeisler et al., 2005; Kumar, 2008), and its off-label uses have shown beneficial effects in improving cognitive function in patients with neuropsychiatric disorders, e.g., Parkinson’s disease, ADHD or PSUD (Peñaloza et al., 2013; Turner et al., 2014). Even though MOD has been suggested as a potential therapeutic agent for the treatment of PSUD (Mereu et al., 2013; Tanda et al., 2021), initial concerns related to its potential abuse liability due to its effects on the central dopaminergic system, akin to those associated with many abused psychostimulants (Jasinski, 2000; Stoops et al., 2005; Volkow et al., 2009). Concerns about its potential for abuse have also been raised by the non-medical use of MOD by healthy individuals to enhance their cognitive function, attention, learning, and memory, in order to improve academic or work-related performance (Fond et al., 2016), leading to a significant debate about potential ethical issues related to a so called “cosmetic neurology” (Cakic, 2009; Sahakian and Morein-Zamir, 2011). However, the increased non-medical use of MOD to potentially improve cognitive performance in school or work settings (Sharif et al., 2021) supports the very low risk, if any, of abuse liability (Kate et al., 2012; Ozturk and Deveci, 2014; Krishnan and Chary, 2015).

### Potential Therapeutic Effects of MOD for PSUD

As summarized in Table 4, clinical studies testing MOD as a potential treatment for PSUD have generated different and sometime inconsistent results.

**TABLE 4:** Results of clinical studies on MOD as a pharmacological therapy for PSUD, including studies on sleep disorders and cognitive dysfunction in PSUD patients.

**Dose/treatment**	**Subjects**		**Experimental info**	**Main effect**	**References**
MOD 300 mg p.o.	Non-drug users	16 healthy, non-drug users	Analysis of behavioral and subjective effects of MOD in comparison to dextroamphetamine and caffeine.	MOD did not produce subjective effects associated with drug abuse liability.	Warot et al. (1993)
MOD 0–400 mg p.o.		6 healthy, non-drug users	Evaluation of behavioral, and subjective effects of treatment	Under active conditions, MOD produced reinforcing effects.	Stoops et al. (2005)
MOD 200–800 mg, methyl-phenidate 45–90 mg, or placebo p.o.	Poly-substances	24 patients with poly-substance abuse history including cocaine	Evaluation of behavioral, and subjective effects of treatment.	MOD failed to produce amphetamine-like subjective effects.	Jasinski (2000)
MOD 200–600 mg p.o.	Cocaine	9 cocaine-dependent patients	Evaluation of the subjective effects of cocaine, MOD, and placebo.	MOD did not produce cocaine-like subjective effects.	Rush et al. (2002a)
MOD 200–600 mg p.o.		6 cocaine-dependent patients	Evaluation of the discriminative, subjective, and cardiovascular effects of cocaine and MOD	MOD did not produce cocaine-like subjective effects.	Rush et al. (2002b)
Placebo or MOD 400 mg p.o. for 8 weeks with CBT		62 cocaine-dependent patients	Urine screen and self-reporting to test cocaine abstinence	MOD (with CBT) improved cocaine dependence more than placebo	Dackis et al. (2005)
MOD 0–800 mg p.o. in combination with 0–40 mg cocaine i.v.		12 cocaine-dependent patients	Analysis of interactions between MOD and cocaine	MOD did not produce any interactions with cocaine	Malcolm et al. (2006)
MOD 0–400 mg p.o.		8 cocaine-dependent patients	Analysis of self-administration of cocaine.	MOD attenuated self-administration of cocaine.	Hart et al. (2008)
Placebo, MOD 200, or 400 mg p.o. for 12 weeks with CBT		210 cocaine-dependent patients	Urine screen and self-reporting to test cocaine abstinence	CBT and MOD effectively increased cocaine non-use days	Anderson et al. (2009)
Placebo or MOD 400 mg p.o. for 16 days.		20 cocaine-dependent patients	Sleep analyses	MOD normalized daily sleep behavior and architecture during cocaine-abstinence	Morgan et al. (2010)
Placebo, MOD 200, or 400 mg p.o. for 8 weeks with CBT		210 cocaine-dependent patients	Urine screen and self-reporting to test cocaine abstinence	No significant effects on abstinence for MOD versus placebo, but trends to significance in male patients	Dackis et al. (2012)
Placebo, or MOD 200 mg		61 cocaine-dependent patients	Urine screen to test cocaine abstinence and neurocognitive tests	MOD improved working memory performance	Kalechstein et al. (2013)
Placebo, MOD 200 mg, escitalopram 20 mg, and MOD 200 mg + escitalopram 20 mg		64 cocaine-dependent patients	Testing of subjective and reinforcing effects as well as cardiovascular measures	MOD blunted the subjective effects of cocaine but escitalopram did not enhance MOD effect	Verrico et al. (2014)
Placebo or MOD 300 mg		15 cocaine-dependent patients	Response effort for cocaine	MOD significantly decreased cocaine choice but only under high cost and alternative condition	Foltin et al. (2016)
Placebo, MOD 400 mg, and CBT		57 cocaine-dependent patients	Sleep analyses	MOD improved sleep and decreased cocaine use	Morgan et al. (2016)
MOD 50–200 mg and CBT	METH	13 METH-dependent patients	Urine screen and self-reporting to test abstinence in HIV gay men	CBT and MOD showed promise with high treatment retention (77%).	McElhiney et al. (2009)
MOD 400 mg and CBT		8 METH-dependent patients	Self-reporting of psychological effects, urine screen for drug abstinence, and cardiovascular effects	No change in cardiovascular or drug abstinence effects	McGaugh et al. (2009)
Placebo or MOD 200 mg p.o. for 10 weeks		80 METH-dependent patients	Urine screen and self-reporting to test METH abstinence, blood pressure, weight gain	Reduction in systolic blood pressure and weight gain, but no improvements on METH dependence	Shearer et al. (2009)
Placebo or MOD 200 mg p.o.		13 METH-dependent patients	Subjective and cardiovascular effects of METH administration and self-administration	No statistical differences in METH use	De La Garza et al. (2010)
Placebo, MOD 400 mg p.o., and CBT for 12 weeks		71 METH-dependent patients	Urine screen to test METH abstinence	MOD did not improve METH dependence more than placebo	Heinzerling et al. (2010)
Placebo or MOD 200 mg p.o.		17 METH-dependent patients	Neuropsychological tests	MOD improved verbal memory recall	Hester et al. (2010)
400 mg MOD or placebo administered p.o.		11 METH-dependent patients	Memory tests	MOD improved working memory	Kalechstein et al. (2010)
Placebo or MOD 200 mg p.o.		24 METH-dependent patients and 17 healthy subjects	Cognitive tests (inhibition, working memory, attention)	Improved attention in patients with high METH use	Ghahremani et al. (2011)
Placebo, MOD 200 mg, or 400 mg p.o. for 12 weeks		210 METH-dependent patients	Urine drug screen for METH non-use week	No effects decrease in METH use. Study compounded by patient compliance issues.	Anderson et al. (2012)
Placebo or MOD 200 mg		18 METH-dependent patients	Evaluation of daytime sleepiness and abstinence	Positive effects on reducing daytime sleepiness associated with decreased METH craving	Mahoney et al. (2012)
Placebo or MOD 200 mg p.o.		19 METH-dependent patients	Self-reporting of sleep, physiological, and craving/withdraw measures	No significant effects of MOD.	Lee et al. (2013)
MOD 200 mg p.o.		80 METH withdrawal patients	Sleep analysis	Improvements in sleep quality	Moosavi et al. (2019)
Placebo or MOD 200 mg p.o.	Nicotine	157 nicotine-dependent patients	Biochemical analysis of nicotine abstinence	No significant effects of MOD	Schnoll et al. (2008)
Placebo, MOD 200–800mg, or methylphenidate 45–90 mg p.o.	Poly-substance users	24 patients with polysubstance abuse history including cocaine	Evaluation of behavioral, and subjective effects of treatment	MOD failed to produce amphetamine like subjective effects.	Jasinski (2000)

*p.o., oral administration; i.v., intravenous; METH, methamphetamine; CBT, cognitive behavioral therapy.*

In an early double-blind, placebo-controlled 8-week study with 62 cocaine dependent patients, MOD 400 mg daily, combined with CBT, significantly improved BE (benzoylecgonine - a cocaine metabolite) negative urine samples over placebo, and significantly increased abstinence rate (3 or more weeks) (Dackis et al., 2005). That study also indicated the safety of MOD administered to cocaine-dependent individuals (Dackis et al., 2005), a finding consistent with previous experimental safety studies that indicated the safety of the co-administration of MOD and intravenous cocaine (Dackis et al., 2003; Malcolm et al., 2006). More recently, another double-blind, placebo-controlled study with cocaine dependent patients (*N* = 94), over an 8-week period, showed that patients treated with 300 mg MOD daily, combined with weekly individual therapy, were significantly more likely to be abstinent than those treated with placebo and weekly individual therapy (Kampman et al., 2015). Furthermore, MOD-treated patients reported significantly lower craving levels compared to those treated with placebo (Kampman et al., 2015). Other experimental human laboratory studies have investigated the potential role of MOD in modulating cocaine’s subjective effects, such as self-reported decreases in ‘good effects,’ ‘stimulation’ and ‘high’ (Malcolm et al., 2006; Hart et al., 2008; McGaugh et al., 2009; Verrico et al., 2014). Further, a decrease in cocaine-associated cardiovascular effects was reported after treatments with both 200 and 400 mg MOD doses, showing an objective physical response, as well as decreased self-administration of high cocaine doses (25 and 50 mg) (Hart et al., 2008).

While the safety of MOD treatments has also been observed in METH-dependent individuals (McGaugh et al., 2009), clinical studies on METH-dependent subjects are less promising than those in cocaine-dependents, although METH studies have been conducted in significantly smaller samples. For example, in a small trial of 13 METH-addicted patients treated with 200 mg of MOD, the authors did not find any significant differences versus placebo, although they reported trends of lowering METH choice by 25% in 3 days of treatment (De La Garza et al., 2010). In a different study, MOD, 200 mg daily, was tested over a 7-day inpatient period on 19 METH abstinent subjects, but no differences, compared to placebo, were found for abstinence, reported craving, or sleep measures (Lee et al., 2013). In another study, METH-dependent patients were assigned to placebo or 200 mg of MOD daily for 10 weeks (Shearer et al., 2009), resulting in no difference in retention and medical adherence between placebo and MOD in self-reported use and urine analysis. The study limitations of relying on self-reported measures and small sample size may confound the results and explain some of the variability among clinical studies for the efficacy of MOD in PSUD (Karila et al., 2010).

### Efficacy of MOD Treatments in Subpopulations of Patients With Cocaine or METH Use Disorder but Without Comorbid Dependences From Other Substances

With inconsistencies plaguing the results of MOD clinical studies, future research should be focused on the specific patient groups who showed beneficial effects from MOD treatment. While Shearer et al. (2009) didn’t find significant differences between placebo and MOD treatments in the entire subjects sample, a *post hoc* analysis showed greatest reductions in METH use compared to placebo with MOD treatments in patients with only METH dependence (removing comorbid opioid dependent patients) (Shearer et al., 2009). MOD didn’t increase the number of non-cocaine use days compared to placebo in a trial of cocaine-dependent patients, but *post hoc* analysis of data found that MOD was superior to placebo in patients without an alcohol codependency (Anderson et al., 2009). Similar outcomes were also reported in a more recent study, 8-week double-blind, placebo-controlled clinical trial in cocaine-dependent subjects without comorbid alcohol dependence, where MOD (300 mg daily) treated patients were more likely to be abstinent from cocaine than patients treated with placebo (Kampman et al., 2015). In another trial on cocaine-dependent patients, no difference was reported between retention or abstinence for MOD treatment compared to placebo, but *post hoc* analysis revealed a significant gender difference with males taking 400 mg MOD showing a greater estimate of abstinence (Dackis et al., 2012).

In a double-blind, placebo controlled trial, MOD, 400 mg daily over 12 weeks, increased retention, while decreasing METH use, depression symptoms, and cravings in those with high METH use and low CBT attendance (Heinzerling et al., 2010). In a different study, it was found that escitalopram, a selective serotonin reuptake inhibitor and commonly prescribed antidepressant, decreased MOD’s effects, raising concern of MOD’s effect on populations of patients treated for depressive disorders alongside drug addiction (Verrico et al., 2014).

Reduced METH use in patients with an abuse diagnosis that included HIV+ participants was found in a 12-week 200 mg MOD study (McElhiney et al., 2009). In the same study, the patients using METH on average 2.2 days per week reported better MOD effects on fatigue due to withdrawal, as well as maintaining abstinence, than patients who used meth 6 days per week (McElhiney et al., 2009). As clinical research on MOD continues, it is increasingly important to study the groups of individuals that do and do not respond to treatment in order to provide critical information toward precision medicine.

### MOD Effects on Sleep Disorders Related to Psychostimulant Use

The relationship between sleep disorders and substance abuse is only loosely understood, but shows some relation with sleep problems reinforcing substance use disorders, as well as substance use leading to sleep disturbances (Angarita et al., 2016). In their review, Angarita et al. (2016) characterized several sleep disturbances produced by alcohol, cocaine, cannabis, and opioid short-term and long-term abstinence, suggesting that substantial research into the effectiveness of sleep agents for addiction treatment is needed. A more recent review links the effects of neurotransmitters on sleep during intoxication and withdrawal from a variety of drugs, but notes the lack of research depth on these neurological interactions and their bearing on drug abuse and dependence (Valentino and Volkow, 2020). Also, gender differences regarding the relationship between drug abstinence and sleep have been described (Coffey et al., 2000; Morgan et al., 2009). A study of short-term METH abstinence found a positive correlation between wanting a nap and craving METH (Mahoney et al., 2012). The study found that a single dose of MOD 200 mg decreased daytime sleepiness, supporting the potential use of MOD as an adjunct treatment for PSUD.

Modafinil has been shown to increase and normalize slow wave sleep to healthy patterns in abstinent cocaine users (Morgan et al., 2010). It was also recently found that while increasing slow wave sleep did not lead to complete, continued abstinence, 400 mg MOD treatment was associated with higher daily rates of abstinence and more consecutive days of abstinence (Morgan et al., 2016). Further, is has been reported that 200 mg MOD improved the sleep quantity and pattern in patients during METH withdrawal (Moosavi et al., 2019).

### MOD Effects on Cognitive Impairment Produced by Psychostimulant Use

Addiction brings changes to the brain beyond the reward pathway. Mental processing dysfunction can hamper rehabilitation attempts and, thus, a drug that can attenuate these risks would be beneficial to the addicted population (Gould, 2010). Cocaine-dependent patients in abstinence showed lower activation compared to healthy controls in areas associated with motor and cognitive functions (Kjome et al., 2010). There have been quite a few studies into MOD’s effects on working memory. In a double-blind, placebo controlled study, it was shown that 400 mg MOD improved working memory in 11 METH-dependent subjects, with poor performance at baseline, after 3 days of treatment (Kalechstein et al., 2010). The same group later showed, in a placebo controlled study, that MOD at 200 mg improved visual and working memory in a group of 61 cocaine-dependent patients, as well as attention and impulsivity, with 5 days of treatment (Kalechstein et al., 2013). While promising, these studies also hold some limitations, in particular the short-term period of treatment and the small samples.

Even though not directly related to PSUD, effects of MOD on performance related to cognitive function were shown in a randomized, double-blind, placebo controlled, crossover study, where 200 mg of MOD administered acutely improved cognitive control in alcohol-dependent patients, but not in the healthy control group (Schmaal et al., 2013). Also, the same researchers showed that administration of 200 mg of MOD improved impulsive decision making in alcohol dependent patients compared to healthy controls (Schmaal et al., 2014). The alcohol dependent group had poor baseline performances compared to the healthy group. This difference could imply that MOD normalizes the brain’s engagement to improve cognition to normal levels in lower performing groups, and the authors suggest that there was likely no room for improvement by MOD in the healthy controls (Schmaal et al., 2014). Further, it has been shown that MOD improved response inhibition in alcohol dependent patients whose initial response was poor (Schmaal et al., 2013). Similar effects related to PSUD were shown in METH-dependent patients in a double-blind, placebo controlled, crossover study, where 200 mg of MOD increased poorly cognitive performance in METH-dependent patients to the same level as the healthy control (Ghahremani et al., 2011). *Post hoc* analysis also revealed that MOD produced larger effects in lower performing participants. Similar findings were also reported in METH-dependent patients where MOD treatments showed larger effects on inhibitory control, processing speed/attention, and motor speed in subject using higher levels of METH compared to those with lower METH usage (Dean et al., 2011). In another study, it was shown that cocaine dependent participants had lower Balloon Analog Risk Task (BART) scores but MOD treated cocaine-dependent participants had higher BART scores, which were comparable to the healthy placebo, showing a normalization of risk taking while on MOD (Canavan et al., 2014).

In a study combining MOD with CBT, it was found that crack cocaine-dependent patients with lower baselines of impulsivity (self-reported) had higher CBT retention and lower crack cocaine use (Nuijten et al., 2016). However, MOD treatment in these patients did not improve CBT retention or outcomes, which is likely as a result of the low MOD adherence during this trial. This study weakness was reported by the same researchers showing that only 10% adherence was reported during a 12 week CBT and MOD trial (Nuijten et al., 2015).

## Beyond MOD: Drug Development of MOD Analogs as Pharmacotherapeutics for PSUD

The effectiveness of MOD as a medication for PSUD has been shown to reach significance in sub-populations of patients without comorbid dependencies from other drugs. In recent years, this important limitation of MOD efficacy has stimulated the development of new structural analogs of MOD to extend therapeutic actions to a broader population and, thus, maximize the effects of the parent drug for use in treatment of PSUD. Some of these novel agents showing atypical DAT blocker properties, have been highlighted in recently published reviews (Newman et al., 2021; Tanda et al., 2021). Among them, some have been shown to bind with high affinity to DAT, and those that promote an inward facing conformation of DAT have shown behavioral and neurochemical preclinical activities different from those of typical abused psychostimulants (Cao et al., 2010, 2016; Okunola-Bakare et al., 2014). Such effects suggest an atypical DAT blocker profile (Keighron et al., 2019a; Newman et al., 2019, 2021; Tanda et al., 2021) and their potential as novel agents for use in the treatment of PSUD.

The effects of MOD analogs on DA neurochemistry have shown varying results (see Table 5). One of the tested analogs, JJC8-016, was unable to stimulate extracellular levels of DA after systemic administration (Zhang et al., 2017; Keighron et al., 2019b), in contrast to other MOD analogs, like JJC8-088 that significantly increased DA levels in a cocaine-like manner, or like JJC8-091 that elicited significant, but less efficacious, increases in DA levels. It is worth noting that the varying effects on stimulation of DA levels were not a result of an altered efficacy as DAT blockers. Indeed, all of these compounds were able to block and reduce DA uptake, an effect highly correlated to their affinity to DAT, as demonstrated by voltammetry studies in rats and mice (Keighron et al., 2019b; Newman et al., 2019). Moreover, their varying ability to enhance the stimulation of elicited DA release in voltammetry studies was unrelated to their affinity for DAT (Keighron et al., 2019b; Newman et al., 2019). These effects once more suggest that compounds that prefer or stabilize an inward facing conformation of DAT would produce limited, if any, cocaine-like effects (Keighron et al., 2019a, b; Giancola et al., 2020; Slack et al., 2020). The same MOD analogs have been tested in behavioral activities related to the reinforcing effects of psychostimulants, and those showing very low stimulation of DA output in microdialysis and voltammetry studies were also among those that produced limited stimulation of ambulatory activities (Keighron et al., 2019a, b; Giancola et al., 2020; Slack et al., 2020). Also, while they did not elicit acquisition or maintenance of self-administration behavior, these MOD analogs blunted cocaine or METH reinforcing and drug-seeking behaviors (Zhang et al., 2017; Tunstall et al., 2018; Newman et al., 2019), suggesting once more that their atypical DAT blocker profile and potential therapeutic activity could be useful as PSUD medications.

**TABLE 5:** Behavioral and neurochemical effects of MOD analogs.

**Agent(s)**	**Dose(s), species**	**Behavioral effects**	**Neurochemical effects**	**References**
JJC8-016	10–30 mg/kg, i.p. RAT	NSE on locomotion when injected alone ↓ Cocaine-induced hyperlocomotion	NSE on stimulation of NAS DA	Zhang et al. (2017)
		Did not induce self-administration ↓ Cocaine self-administration		
		↓ Reinstatement of cocaine seeking behavior		
	3–100 mg/kg, i.p. MICE	NSE on ambulatory behavior	NSE on stimulation of NAS DA	Keighron et al. (2019b)
			NSE on stimulation of evoked DA release in NAS	
			↓ NAS DA clearance	
	10–30 mg/kg, i.p. RAT	↓ METH self-administration following both short and long access to drug		Tunstall et al. (2018)
JJC8-088	1–56 mg/kg, i.p. RATS	↓ Cocaine self-administration	↑ NAS DA efflux	Newman et al. (2019)
		↑ Optical intracranial self-stimulation	↑ Evoked DA release in the NAS	
		NSE on cocaine PR breakpoints	↓ NAS DA clearance	
	3–30 mg/kg, i.p. RAT	NSE on METH self-administration following both short and long access to drug		Tunstall et al. (2018)
	3–56 mg/kg, i.p. MICE	↑ Ambulatory behavior	↑ NAS DA efflux	Keighron et al. (2019b)
			↑ Evoked DA release in the NAS	
			↓ NAS DA clearance	
JJC8-091	1–56 mg/kg, i.p. RATS	NSE on cocaine FR self-administration	↑ NAS DA efflux	Newman et al. (2019)
		↓ PR breakpoints for cocaine	NSE on evoked NAS DA release	
		↓ Cocaine primed reinstatement	↓ NAS DA clearance	
		↓ Optical intracranial self-stimulation		
	10–56 mg/kg, i.p. RAT	↓ METH self-administration following both short and long access to drug		Tunstall et al. (2018)
	3–100 mg/kg, i.p. MICE	NSE on ambulatory behavior	↑ NAS DA efflux	Keighron et al. (2019b)
			NSE on evoked NAS DA release	
			↓ NAS DA clearance	

*NSE, not a significant effect; METH, methamphetamine; FR, fixed ratio; PR, progressive ratio; i.p., intraperitoneal; ↑, increase; ↓, decrease.*

## Conclusion

Modafinil is clinically approved for narcolepsy and other sleep disorders (Bastoji and Jouvet, 1988; Broughton et al., 1997; US Modafinil in Narcolepsy Multicenter Study Group, 1998, 2000), but its off-label use for treatment of several psychiatric disorders has been repeatedly reported (Ballon and Feifel, 2006; Peñaloza et al., 2013; Turner et al., 2014). During the last two decades, there have been several preclinical and clinical studies that suggested potential efficacy of MOD as a treatment for PSUD, but also contrasting results from other studies which limited its progression (Lee et al., 2013; Schmitz et al., 2014). Among the positive results, it is interesting to note that after many years of clinical use, there are only a few reports of abuse in MOD-treated patients (Kate et al., 2012; Ozturk and Deveci, 2014; Krishnan and Chary, 2015), a result in agreement with clinical and preclinical studies showing its limited, if any, potential for abuse (Jasinski, 2000; Deroche-Gamonet et al., 2002; Myrick et al., 2004; Food and Drug Administration, 2007; Vosburg et al., 2010). On the other hand, disappointing results of clinical trials testing MOD as a treatment for PSUD have been obtained in the general population of drug-dependents. However, based on results from several of those reports, positive treatment outcomes have been found when the population sample included only subjects with psychostimulant dependency, without concurrent alcohol or other drug dependencies (Anderson et al., 2009; Shearer et al., 2009; Kampman et al., 2015). These studies underscore the importance of pursing personalized treatment approaches for PSUD, similarly to other medical disorders (Hamburg and Collins, 2010; Schork, 2015). It is clear that the complexity of PSUD, the huge differences in how PSUD develops among the population, and the presence of many other individual, genetic, or environmental variables, suggest it is unlikely that there will ever be a “silver bullet” medication to treat all individuals with PSUD. Thus, personalized medicine approaches, together with behavioral cognitive treatments, might be the most effective path to reduce the harm produced by PSUD. While MOD has been shown to improve several emerging pathological conditions related to psychostimulant use, i.e., dependence, sleep, and cognitive impairments, its overall limited success has triggered medicinal chemistry research toward discovery of structural analogs of MOD, that might hold more robust efficacy in PSUD. In conclusion, while MOD could be an effective pharmacological treatment already available for subpopulations of individuals suffering from PSUD, new pharmacological tools derived from MOD show promising preclinical efficacy and could help to provide more efficacious future treatment opportunities for PSUD.

## Author Contributions

All authors contributed to the manuscript and approved the submitted version.

## Conflict of Interest

The authors declare that the research was conducted in the absence of any commercial or financial relationships that could be construed as a potential conflict of interest. The reviewer TK declared a past co-authorship with one of the authors, AN, to the handling editor.

## Abbreviations

ADHD: attention deficit hyperactivity disorder
CNS: central nervous system
CPP: conditioned place preference
CBT: cognitive behavioral therapy
DA: dopamine
DAT: dopamine transporter
DS: dorsal striatum
ICSS: intracranial self-stimulation
MOD: modafinil
METH: methamphetamine
NAcc: nucleus accumbens
NAS: nucleus accumbens shell
NE: norepinephrine
NET: norepinephrine transporter
PET: positron emission tomography
PFC: prefrontal cortex
PSUD: psychostimulant use disorder
R-MOD: (R)-enantiomer of modafinil
VMAT2: vesicular monoamine transporter
VS: ventral striatum.
